# Antagonizing circRNA_002581–miR-122–CPEB1 axis alleviates NASH through restoring PTEN–AMPK–mTOR pathway regulated autophagy

**DOI:** 10.1038/s41419-020-2293-7

**Published:** 2020-02-13

**Authors:** Xi Jin, Jianguo Gao, Ruoheng Zheng, Mosang Yu, Yue Ren, Tianlian Yan, Yue Huang, Youming Li

**Affiliations:** 1 Department of Gastroenterology, the First Affiliated Hospital, School of Medicine, Zhejiang University, 310003 Hangzhou, China; 2 School of Clinical Medicine, Hangzhou Medical College, 310053 Hangzhou, China; 3 School of Medicine, Zhejiang University, 310058 Hangzhou, China

**Keywords:** Macroautophagy, Non-alcoholic fatty liver disease

## Abstract

Circular RNAs (circRNAs) have been shown to play critical roles in cancer biology, but their functions in nonalcoholic steatohepatitis (NASH) remain unexplored. Full length of circRNA_002581 was amplified and sequenced, followed by RNA immunoprecipitation, RNA-Fluorescence in Situ Hybridization and dual luciferase reporter gene analysis to confirm the existence of the circRNA_002581–miR-122–CPEB1 regulatory axis in vitro. CircRNA_002581 knockdown was used to study its roles in high concentration of free fatty acids-induced NASH-like cell model and a methionine and choline deficiency (MCD) diet-induced NASH mice model. Autophagy flux and related potential PTEN–AMPK–mTOR pathway were tested by western blot. CircRNA_002581 overexpression significantly relieved the inhibitory role of miR-122 on its target CPEB1 by sponging miR-122. CircRNA_002581 knockdown markedly attenuated lipid droplet accumulation, reduced the levels of alanine aminotransferase (ALT), aspartate aminotransferase (AST), pro-inflammatory cytokines, apoptosis, H_2_O_2_, and increased ATP level in both mice and cellular models of NASH. Mechanistically, circRNA_002581 interference significantly rescue the defective autophagy evidenced by increased autophagosome number, upregulated LC3-II/I level, and decreased p62 level. Further chloroquine-mediated total autophagy inhibition antagonizes the protective effect of circRNA_002581 knockdown. Finally, CPEB1–PTEN–AMPK–mTOR pathway is shown to link the autophagy and circRNA_002581 knockdown-mediated NASH alleviation. CircRNA_002581–miR-122–CPEB1 axis actively participates in the pathogenesis of NASH through PTEN–AMPK–mTOR pathway-related autophagy suppression. Targeting circRNA_002581 is a potential therapeutic strategy for NASH through partial autophagy restoration.

## Introduction

Nonalcoholic steatohepatitis (NASH) is considered as a pivotal stage in nonalcoholic fatty liver disease (NAFLD), where an increasing number of ensuing liver cirrhosis, end-stage liver disease and even hepatocellular carcinoma (HCC) has been observed, becoming a big threat to human health^1^. Furthermore, extra-hepatic diseases are also increased in NASH stage, such as cardiovascular disease^2^. Additionally, steatohepatitis is the driving force for fibrosis development that is a vital pre-stage of liver cirrhosis and predicts mortality of NAFLD^3^. Therefore, exploring the mechanism, diagnosis, and treatment of NASH is of both theoretical and clinical importance while the mechanism of research serves as the basic step. Though “two-hit” hypothesis has been well acknowledged^4^, recent studies advocated the convergent impacts of the environment, microbiome, metabolism, and genetic risk factors. Nevertheless, the inter-relationship among those factors, the detailed mechanisms and the upstream regulatory factors/downstream effectors are still vague.

Circular RNA (circRNA) is one type of rediscovered endogenous noncoding RNA, forming covalently closed continuous loop structure through specific splicing method and is considered as the major subtype in gene transcription^5^. One important peculiarity of circRNA is its “microRNA (miRNA) sponge” function, where it can efficiently bind and inhibit miRNA activity, further influence downstream mRNA expression and finally participate in various diseases^6^. For instance, circRNA Cdr1as was reported to regulate insulin transcription and secretion in islet cells via binding to miR-7 and further influence its downstream target mRNAs expression^7^. Nevertheless, current reports on the circRNA in NAFLD have been rarely reported, including the metformin-induced changes of the coding transcriptome and non-coding RNAs in NAFLD mice^8^ and the prevention of hepatocyte lipid peroxidation and steatosis by circRNA_0046367^9^. Therefore, circRNA function in NAFLD is a novel research arena and calls for further intensive study.

Based on the “sponge” effect of certain circRNA, it is important to find its downstream miRNA–mRNA for function study, where our previously identified circRNA profile and circRNA–miRNA–mRNA network in NASH has provided data reservoir^10^. Among our identified significantly changed mRNAs, the cytoplasmic polyadenylation element-binding protein 1 (CPEB1) has attracted our interests. CPEB1 is an mRNA-binding protein that controls cytoplasmic polyadenylation-induced translation by interacting with the 3′ UTR cis-acting cytoplasmic polyadenylation element (CPE) and three regulatory proteins including Gld2, PARN, and Maskin^11^. Except for the routine regulation activity on germ cell development, synaptic plasticity, and cellular senescence, the increased CPEB1 level was found to induce the pathologic angiogenesis in chronic liver disease^12^. Moreover, its homolog CPEB4 was identified to mediate a translational response to counteract hepatic steatosis under endoplasmic reticulum (ER) stress^13^. All these evidences support the potential role of CPEB1 in NASH and needs further investigation.

Our previous study indirectly advocated the positive regulation of circRNA_002581 on CPEB1 through sponging microRNA 122 (miR-122) by bioinformatics prediction and partial quantitative real-time PCR (qRT-PCR) verification^10^. Since miR-122 is the most frequent miRNA in adult liver and a key factor and therapeutic target in liver disease^14^, this character has put heavy weight on the potential effect of circRNA_002581–miR-122–CPEB1 axis in the pathogenesis of NASH. During searching for its downstream pathophysiological effector, autophagy, an important process of degrading incorrect folded proteins and injured organelles for keeping cell and tissue homeostasis^15^, has attracted our attention. On one hand, previous research showed that the autophagy level was decreased in NASH patients and its activation could decrease hepatic steatosis^16^, indicating the role of autophagy in NASH. On the other hand, mammalian target of rapamycin (mTOR) is the key inhibitor of autophagy and regulated by phosphatase and tensin homolog (PTEN)-promoted AMP-activated protein kinase (AMPK) phosphorylation^17,18^. Further study showed the activation of CPEB1 deletion on PTEN expression^19^, bringing the circRNA_002581–miR-122–CPEB1 axis between NASH phenotype and autophagy.

Therefore, in this study, we aimed to provide direct evidence of the existence of the circRNA_002581–miR-122–CPEB1 axis, test the effect of antagonizing circRNA_002581 on NASH severity, investigate whether autophagy restoration is the downstream effector and finally, find whether the PTEN–AMPK–mTOR pathway acts as the bridge between such axis and autophagy change.

## Results

### CircRNA_002581 full length amplification, sequencing, and bioinformatics analysis

**Fig. 1:**
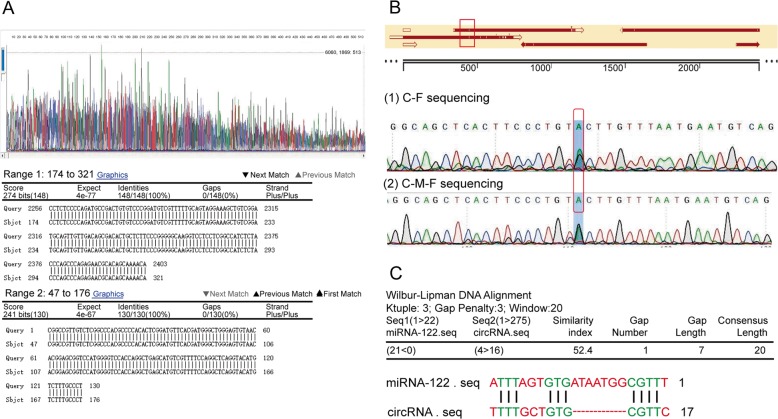
Amplification, sequencing and blasting the spliced and full length of circRNA_002581 retrieved from the livers of NASH mice with known NCBI data. **a** The upper column showed the PCR-sequencing graph of spliced length of circRNA_002581; the lower column showed 100% matching of such spliced length of 275 bp with known NCBI data of circRNA_002581. **b** The upper column showed the results of sequence blasting, where there is a spot mutation at position 447 (red box); the lower column further showed that the double peak at position 447, indicating the existence of heterozygote. **c** Bioinformatics analysis predicted potential-binding site between circRNA_002581 and miR-122.

The core length of circRNA_002581 was firstly amplified with two backward designed primers, with 100% matching to the 275 spliced sequence length from NBCI base (Fig. 1a). After additional five primers amplification of the full-length sequence of circRNA_002581, the results showed a G-A point mutation at position 447, which is out of the spliced sequence area (Fig. 1b). Such mutation may be caused by the occurrence of NASH. Further bioinformatics analysis predicted one binding site between circRNA_002581 and miR-122 (Fig. 1c).

### Confirming the existence of circRNA_002581–miR-122–CPEB1 regulatory axis

**Fig. 2:**
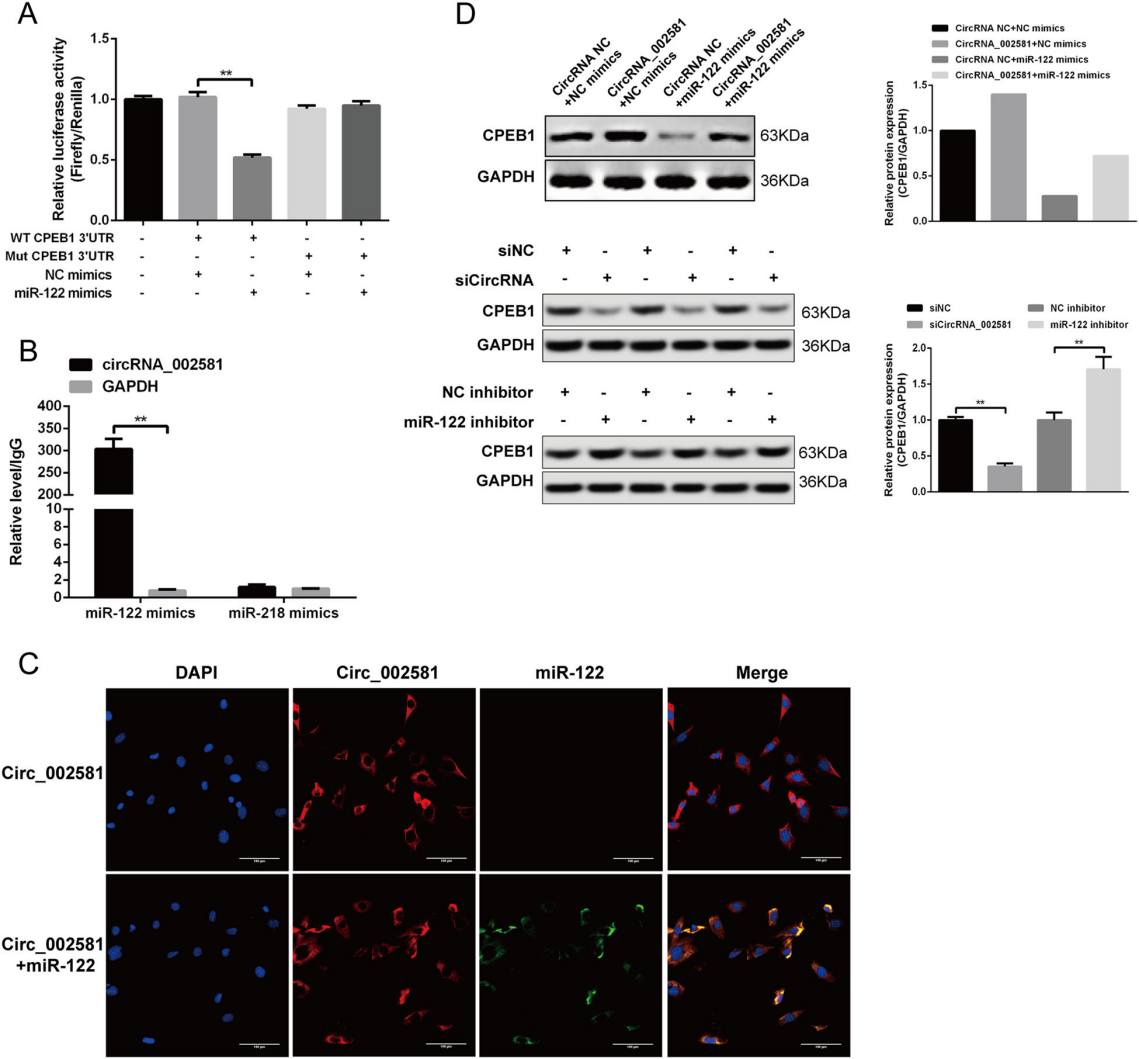
Confirming the existence of circRNA_002581–miR-122–CPEB1 axis. **a** Dual luciferase reporter gene analysis showed the direct binding between miR-122 and the 3′UTR wild type of CPEB1. The miRNA negative control mimics and the 3′UTR mutant type of CPEB1 have lost their binding capacity for each other. **b** RIP result showed circRNA_002581 in NIH3T3 cell lysis was pulled down and detected by qPCR with selected primer, indicating the existence of binding between circRNA_002581 and miR-122, where miR-218 was used as negative miRNA control and GAPDH was applied as control. **c** RNA FISH showed the co-localization of miR-122 (green) and circRNA_002581 (red) in the cytoplasm of HEK-293T cells. The blue spot is the nucleus dyed by DAPI (scale bar: 100 μm). **d** Western blot showed CPEB1 level was decreased when transfecting HEK-293T cells with miR-122 mimics than with NC mimics. Further co-transfecting circRNA_002581 could antagonize the inhibitory effect of miR-122 mimics on CPEB1 protein level (upper panel); CPEB1 protein level was significantly downregulated when transfected with siCircRNA_002581 in NCTC-1469 cells but significantly upregulated when transfected with miR-122 inhibitor (middle and lower panels). Error bars represent the SD. **p < 0.01.

The successful establishment of circRNA_002581 and miR-122 overexpression plasmid (termed as miR-122 mimics thereafter) was shown by qRT-PCR after transfecting HEK-293T cell (Supplementary Fig. 1). The direct regulation of miR-122 on CPEB1 was confirmed by dual luciferase reporter gene analysis. As shown in Fig. 2a, miR-122 could significantly decrease the relative light unit when co-transfected with wild type 3′UTR of CPEB1, while the other groups including miRNA negative control (NC) mimics and mutant type 3′UTR of CPEB1 took no effect. The direct regulation of circRNA_002581 on miR-122 was verified by joint RNA immunoprecipitation (RIP) and RNA-fluorescence in situ hybridization (FISH). As shown in Fig. 2b, the RIP result showed that there was no circRNA_002581 detected in control group (miR-218 mimics). However, in miR-122 mimics group, the circRNA_002581 could be amplified by the 1st and 16th primers (one representative result was shown), indicating the existence of binding site between circRNA_002581 and miR-122. As illustrated in Fig. 2c, further RNA-FISH indicated the co-localization between circRNA_002581 and miR-122 in the cytoplasm of HEK-293T cell, indirectly supporting the physical interaction between these two molecules. To further support the existence of circRNA_002581–miR-122–CPEB1 pathway, we tested the indirect regulation of circRNA_002581 on CPEB1 expression. As shown in Fig. 2d, miR-122 markedly decreased the protein level of CPEB1, while over-expressing circRNA_002581 could markedly relieve the inhibitory role of miR-122 on CPEB1; in addition, knockdown of the endogenous expression of circRNA_002581 significantly reduced the protein expression of CPEB1, while inhibition of endogenous miR-122 could significantly upregulate CPEB1 protein expression.

### Effect of antagonizing circRNA_002581 expression in high concentration of free fatty acids (HFFA)-induced NASH-like cell model

**Fig. 3:**
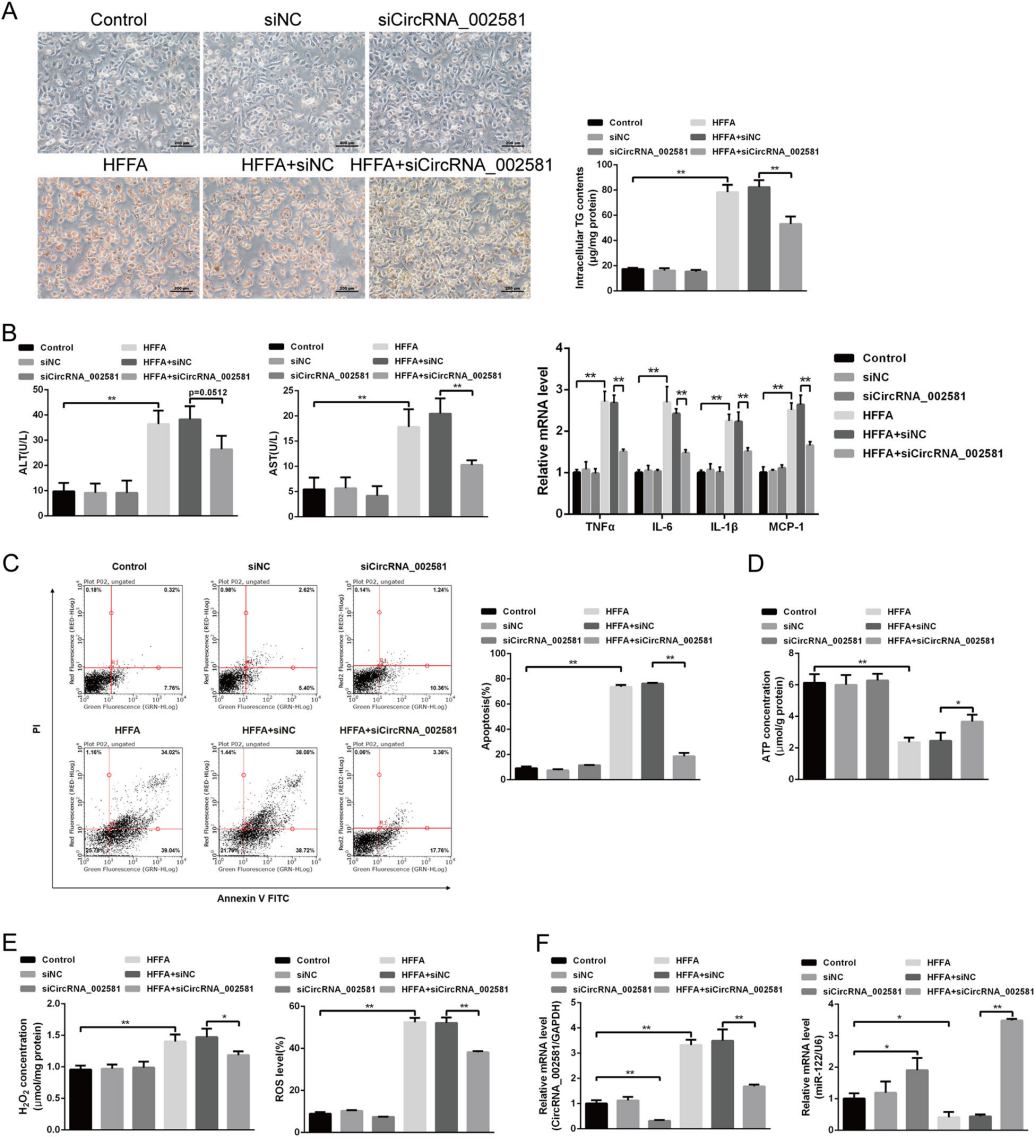
Effect of antagonizing circRNA_002581 in HFFA-induced NASH-like cell model. NCTC-1469 cells were transfected with scrambled siRNA (negative control) or circRNA_002581 siRNA and then treated with or without HFFA (1 mM) for 72 h. **a** Representative images of Oil Red O staining of NCTC-1469 cells (scale bar: 200 μm) and intracellular TG contents. **b** ALT and AST levels in culture supernatants; and relative mRNA levels of TNFα, IL-6, IL-1β, and MCP-1 by quantitative real-time PCR. **c** Apoptotic cells were identified by flow cytometry. **d** ATP concentration in NCTC-1469 cells. **e** H_2_O_2_ concentration and ROS level in NCTC-1469 cells. **f** Relative mRNA levels of CircRNA_002581 and miR-122 by quantitative real-time PCR. Error bars represent the SD. *p < 0.05; **p < 0.01.

Since the expression of circRNA_002581, miR-122, and CPEB1 may vary in different cell lines, we initially chose two mice normal hepatocytes AML-12 and NCTC-1469 to establish the NASH cell like model. Though triglyceride (TG), circRNA_002581 and miR-122 levels were all significantly changed in both two-cell lines, the CPEB1 level was only significantly increased in NCTC-1469 cell (Supplementary Fig. 2). Therefore, NCTC-1469 cell was chosen to establish NASH cell like model. We found obviously increased red lipid droplet accumulation and intracellular TG contents in HFFA-treated group after 72 h HFFA culture, while small interfering RNA (siRNA) targeting circRNA_002581 could significantly decrease such accumulation (Fig. 3a). As shown in Fig. 3b–e, the levels of ALT, AST, tumor necrosis factor α (TNFα), interleukin-6 (IL-6), interleukin-1β (IL-1β), monocyte chemoattractant protein-1 (MCP-1), apoptosis, H_2_O_2_, and reactive oxygen species (ROS) were significantly increased, while ATP level significantly decreased in HFFA-induced NASH cell-like model (all *p* < 0.05). The knockdown of circRNA_002581 could significantly antagonize those changes. Further significantly increased circRNA_002581 and decreased miR-122 levels were identified in HFFA-induced NASH cell-like model while decreasing circRNA_002581 could antagonize those changes (Fig. 3f). To clarify the role of miR-122 in antagonizing circRNA_002581-induced protective effect, we used miR-122 inhibitor and found that inhibition of miR-122 reversed knockdown of circRNA_002581-ameliorated, HFFA-induced intracellular TG levels and inflammation levels (Supplementary Fig. 3).

### Effect of antagonizing circRNA_002581 expression in methionine and choline deficiency (MCD)-induced NASH mice model

**Fig. 4:**
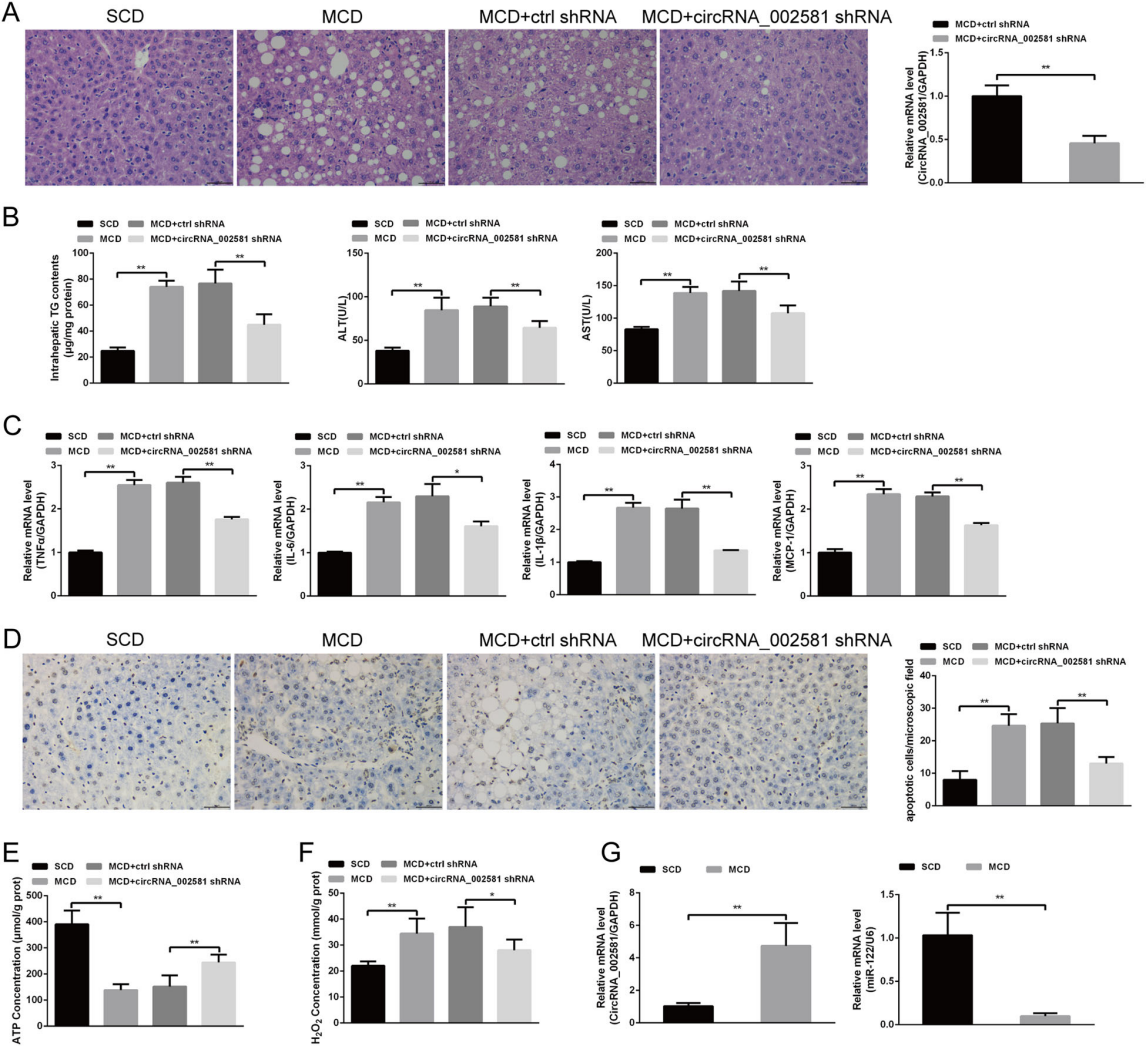
Effect of antagonizing circRNA_002581 in MCD-induced NASH mice model. BALB/c mice were fed with SCD or MCD, and two groups of MCD-fed mice were, respectively, given tail vein injection of 5 × 10^8^ IU control or circRNA_002581 shRNA packaged Lentivirus once per 5 days from the beginning of experiment and were sacrificed after 4 weeks feeding. **a** Representative images of H&E staining of livers (scale bar: 100 μm) and the knockdown efficiency of shRNA targeting circRNA_002581 by quantitative real-time PCR. **b** intrahepatic TG contents, serum ALT, and AST levels. **c** Relative hepatic mRNA levels of TNFα, IL-6, IL-1β, and MCP-1 by quantitative real-time PCR. **d** Apoptotic cells in the liver were detected by TUNEL assay (scale bar: 100 μm). **e** ATP concentration in the liver. **f** H_2_O_2_ concentration in the liver. **g** Relative hepatic mRNA levels of circRNA_002581 and miR-122 by quantitative real-time PCR. Error bars represent the SD. *p < 0.05; **p < 0.01.

The NASH animal model was successfully established after MCD induction, as shown by lipid droplet accumulation with H–E staining (Fig. 4a) and increased TG contents (Fig. 4b). We identified significantly increased ALT, AST in the serum level, and mRNA level of pro-inflammatory cytokines (TNFα, IL-6, IL-1β, MCP-1) in the liver of MCD-induced NASH model (Fig. 4b, c). Further Terminal deoxynucleotidyl transferase dUTP Nick-End Labeling (TUNEL) analysis showed significantly increased apoptosis degree in NASH model (Fig. 4d). The results also identified significantly increased H_2_O_2_ levels but decreased ATP level in the liver tissue of MCD-fed mice (Fig. 4e, f). As illustrated in Fig. 4g, the mRNA level of circRNA_002581 was markedly upregulated, while the mRNA level of miR-122 was significantly downregulated in the livers of MCD-induced NASH mice model. After successful circRNA_002581 shRNA injection, the knockdown efficiency of short hairpin RNA (shRNA) targeting circRNA_002581 was ~54% (Fig. 4a). The severity of NASH in MCD + circRNA_002581 shRNA group was alleviated when comparing with MCD and MCD + ctrl shRNA groups, as shown by the significantly decreased hepatocellular lipid accumulation and inflammation (see NAS score in Supplementary Table 1), intrahepatic TG contents (Fig. 4b), levels of apoptosis degree, ALT, AST, TNFα, IL-6, IL-1β, MCP-1, and H_2_O_2_ as well as the significantly restored ATP level (Fig. 4).

### Autophagy suppression in NASH and partial re-activation after circRNA_002581 antagonization

**Fig. 5:**
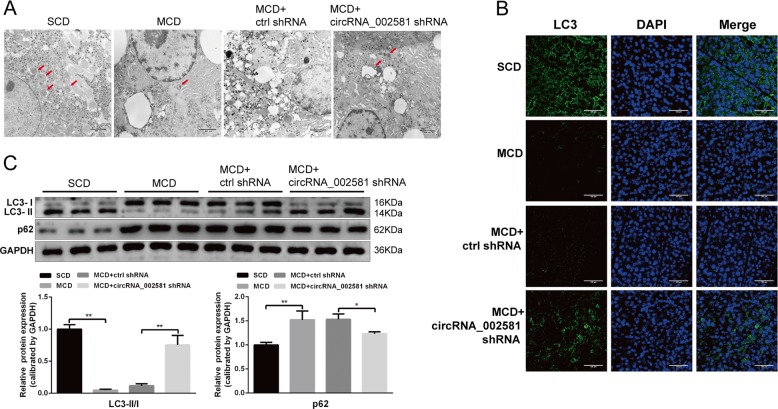
Effect of antagonizing circRNA_002581 on autophagy in MCD-induced NASH mice model. **a** Representative TEM images of autophagosome (pointed with red arrow) in the livers (scale bar: 800 nm). **b** Confocal microscopy analysis for autophagosome by immunofluorescent staining of LC3 in the livers (scale bar: 100 μm). **c** Western blot analyses of hepatic LC3-II/I and p62. Error bars represent the SD. *p < 0.05; **p < 0.01.

In in vivo level, transmission electron microscope (TEM) showed that the number of autophagosome in the liver of MCD-fed mice was decreased but obviously increased in MCD mice with circRNA_002581 shRNA injection (Fig. 5a). Further immunofluorescence result showed that LC3 level was decreased in MCD group while circRNA_002581 interference could partially antagonize such change (Fig. 5b). Additional western blot demonstrated the significantly increased levels of p62 and decreased levels of LC3-II/I in MCD group. Those changes were significantly antagonized by circRNA_002581 knockdown (Fig. 5c).

**Fig. 6:**
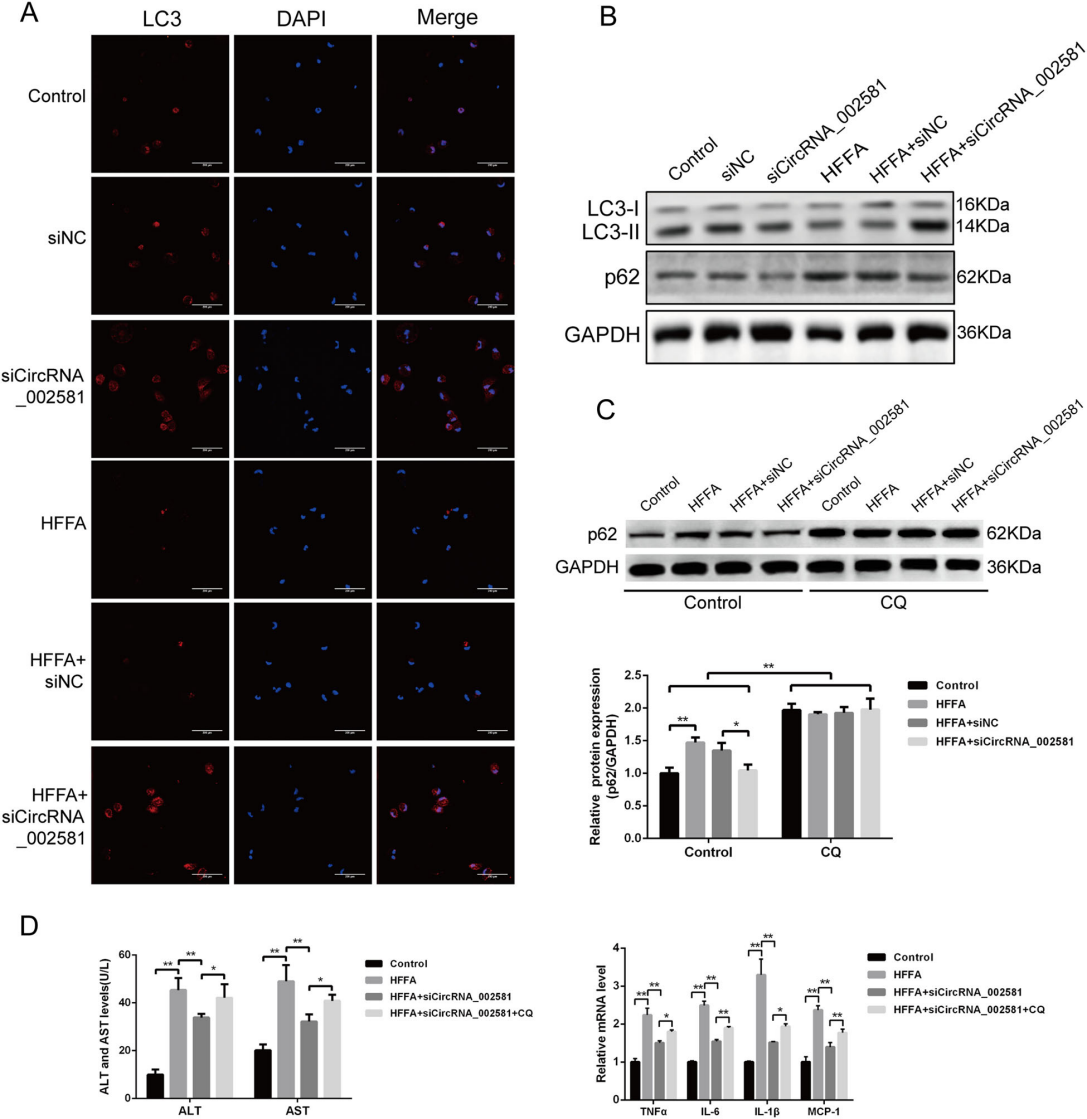
Involvement of autophagy in circRNA_002581 knockdown mediated alleviation of HFFA-induced NASH phenotype in vitro. **a** Confocal microscopy analysis for autophagosome by immunofluorescent staining of LC3 in NCTC-1469 cells (scale bar: 200 μm). **b** Western blot analyses of LC3-II/I and p62 in NCTC-1469 cells. **c** NCTC-1469 cells were pretreated with or without CQ (20 μM), and followed by incubation with the combination of HFFA, scrambled siRNA (negative control) and circRNA_002581 siRNA for 72 h. p62 level in NCTC-1469 cells was detected by western blot. **d** NCTC-1469 cells were incubated with different combination of HFFA, circRNA_002581 siRNA, and CQ (20 μM). ALT and AST levels in culture supernatants were detected. Relative mRNA levels of TNFα, IL-6, IL-1β, and MCP-1 by quantitative real-time PCR. Error bars represent the SD. *p < 0.05; **p < 0.01.

In vitro analysis also advocated the same trends of LC3 immunofluorescence change and p62, LC3-II/LC3-I expression in HFFA-induced NASH-like cell model with and without circRNA_002581 interference (Fig. 6a, b). Further autophagic flux test showed that p62 levels were significantly increased in all chloroquine (CQ)-treated groups than their counterparts in CQ-untreated groups. Besides, previously observed circRNA_002581 antagonization mediated p62 declination was totally abolished by CQ pre-treatment (Fig. 6c). Finally, though the steatosis phenotype of NASH-like cell was not influenced, its previously partially alleviated inflammation phenotype by circRNA_002581 knockdown was, in different degrees, antagonized by CQ-induced total autophagy inhibition (Fig. 6d). To sum up, our results indicated that autophagy actively participated in NASH and acted as, at least, one of the important downstream pathophysiological targets of circRNA_002581 knockdown-guided anti-NASH effects.

### PTEN–AMPK–mTOR pathway linking the regulatory effect of circRNA_002581–miR-122–CPEB1 axis on autophagy

**Fig. 7:**
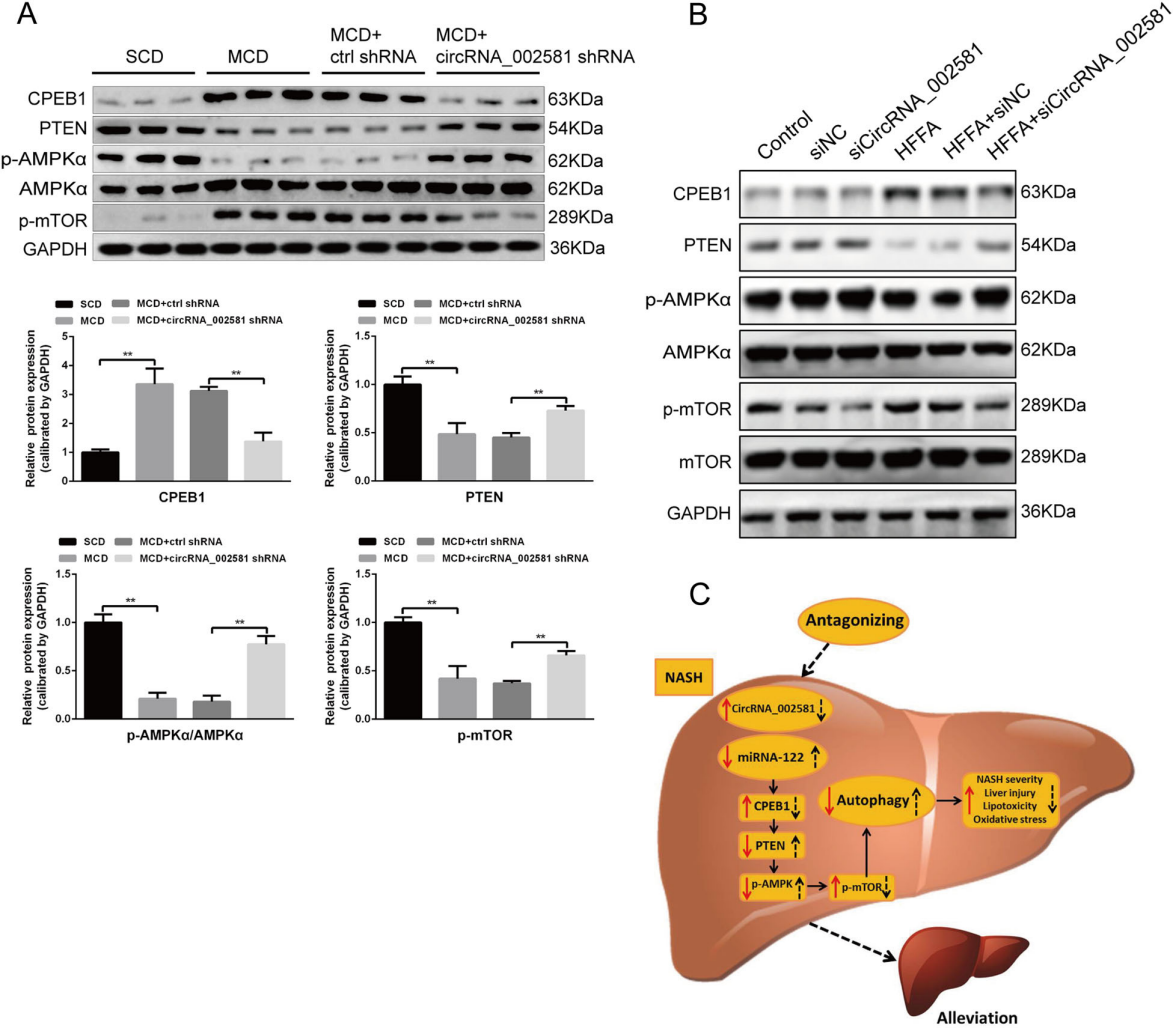
CPEB1–PTEN–AMPK–mTOR pathway potentially participated in the effect of circRNA_002581 on autophagy. **a** Western blot analyses of CPEB1, PTEN, p-AMPKα, AMPKα, and p-mTOR in the livers. **b** Western blot analyses of CPEB1, PTEN, p-AMPKα, AMPKα, p-mTOR, and mTOR in NCTC-1469 cells. **c** A proposed model depicting the mechanism that antagonizing CircRNA_002581 alleviates NASH progression. In NASH, elevated CircRNA_002581 acts as the sponge of miR-122 and then upregulates expression of its target gene CPEB1, which may subsequently impair autophagy via PTEN–AMPK–mTOR pathway, thereby aggravating NASH progression (red solid arrow). Conversely, antagonizing CircRNA_002581 attenuates miR-122 inhibition then downregulates CPEB1 expression which results in partial autophagy restoration through PTEN–AMPK–mTOR pathway, ultimately contributing to NASH alleviation (black dashed arrow). Error bars represent the SD. **p < 0.01.

Based on our previously established circRNA_002581–miR-122–CPEB1 axis and its effect in NASH, the literature reported CPEB1–PTEN–AMPK–mTOR pathway and the inhibition effect of mTOR on autophagy, we started to investigate whether this pathway could explain the change of autophagy in NASH and the effect of circRNA_002581 knockdown on NASH alleviation through influencing autophagy. Briefly, western blot demonstrated the significantly increased levels of CPEB1 and p-mTOR, as well as the significantly decreased levels of PTEN and p-AMPKα/AMPKα in MCD group. Those changes were significantly antagonized by circRNA_002581 knockdown (Fig. 7a). Further in vitro analysis also advocated the same trends of protein expression in NASH with and without circRNA_002581 interference, as in in vivo results (Fig. 7b).

## Discussion

NASH deserves in-depth study for its high occurrence rate, unclear mechanisms and clinical importance. CircRNA was reported to participate in liver regeneration^20^ and act as the molecular marker and therapeutic target of liver cancer^21^. CircRNA_002581 (also named circ_0001351 in circBase) is located at the exonic area of mouse genome in chromosome 5 from position 66,753,956 to 66,756,359, with full length of 2403 bp and spliced length of 275 bp. Its corresponding linear form is NM_001201414, encoding protein amyloid beta precursor protein-binding family B member 2 (APBB2). Genetic association of APBB2 with late onset Alzheimer disease was reported in 2005^22^. According to the RIP result and bioinformatics analysis, there are one binding sites between circRNA_002581 and miR-122. It is not surprising for the relatively fewer binding sites in this study than the over 70 binding sites between ciRS-7 and miR-7^23^, since most natural miRNA sponges and competing endogenous RNAs contain only 1–2 miRNA-binding sites^24^. In addition, the RNA-FISH assay in vitro showed that circRNA_002581 was co-localized with miR-122 in the cytoplasm, which is in agreement with the interaction between circRNA_002581 and miR-122.

Accumulated studies indicate that miRNAs play important roles in various liver diseases^25,26^. MiR-122 level was significantly decreased in NASH patients^27^ while both liver-specific and germline miR-122 knockout mice developed hepatic steatosis in early adult life, then progressing to NASH, liver fibrosis, and eventually HCC with age^28,29^. In addition, inhibition of miR-122 leads to increased hepatic TG accumulation and suppressed liver fat oxidation^30^. Collectively, these studies demonstrate that miR-122 plays an important protective role in the progression of NAFLD. In agreement with our previous findings that miR-122 was the predicted downstream target of circRNA_002581 and upstream regulator of CPEB1^10^, in this study, we found that (1) miR-122 is a direct regulator of CPEB1 and target of circRNA_002581 as evidenced by dual luciferase reporter gene analysis, RIP and RNA-FISH, (2) overexpression of circRNA_002581 could significantly attenuate miR-122-mediated CPEB1 downregulation in vitro, (3) knockdown of the endogenous expression of circRNA_002581 significantly reduced the protein expression of CPEB1, while inhibition of endogenous miR-122 had the opposite effect, (4) circRNA_002581 knockdown could significantly abolish CPEB1 upregulation both in NASH animal and cell model. Taken together, these findings suggest that CircRNA_002581–miR-122–CPEB1 pathway is involved in the pathogenesis of NASH.

The pathogenesis of NASH is still unclear and now is characterized by the ‘multiple parallel hits’ hypothesis including inflammatory cytokines, oxidative stress, lipotoxicity-induced lipoapoptosis, ER stress, and mitochondrial dysfunction^31^. In the present study, we found that circRNA_002581 knockdown markedly attenuated hepatic lipid accumulation and inflammation, hepatocyte apoptosis and oxidative stress in NASH cell and mice model (Figs. 4, 5). Although several recent studies have reported that circRNAs play an important role in the stage of simple steatosis^9,32,33^, it is the first time the current study demonstrates that circRNA participates in the progression of NASH and circRNA_002581 may serve as a novel therapeutic target for the treatment of NASH.

Numerous studies have addressed that defective autophagy function contributes to the progression of NASH due to abnormal hepatic lipid metabolism, insulin resistance, enhanced inflammation, and increased cell death^34–36^. In present study, we reconfirmed the suppression of autophagy in NASH and found that circRNA_002581 interference significantly rescued the defective autophagy in both NASH mice and cell model, as evidenced by increased autophagosome number, upregulated LC3-II/I level and decreased p62 level (Figs. 5 and 6a, b). Though the mutual transformation between LC3-II and LC3-I could indirectly reflect the status of autophagy flux^37^, the phenomenon of decreased LC3-II level in NASH may reflect both autophagy suppression and over activation, causing the increased consumption of LC3-II in autophagolysosome degradation. Therefore, CQ, a well-known autophagy inhibitor, was pre-added and we found the significantly increased p62 levels in all CQ-treated groups with total abolishment of previous observed autophagy status in NASH with/without circRNA_002581 knockdown (Fig. 6c). The ensuing experiment showed that total autophagy inhibition by CQ could antagonize the therapeutic effect of circRNA_002581 knockdown on NASH phenotype in in vitro level, as evidenced by several significantly re-increased inflammatory markers (Fig. 6d). Therefore, we postulate that autophagy is the key pathophysiological target of NASH and the therapeutic effector of circRNA_002581.

To gain insight into the potential linking pathway between circRNA_002581 and autophagy, we investigated the protein levels of well-documented autophagy regulators and observed that circRNA_002581 knockdown reversed the upregulation of CPEB1 and p-mTOR, the downregulation of PTEN and p-AMPKα both in NASH mice and cell model. In support of these findings, studies have demonstrated that p-AMPK positively regulate autophagy through inhibition of p-mTOR, while PTEN promotes autophagy through upregulation of the p-AMPK^17,18^. Furthermore, a study has revealed that CPEB1 directly downregulates the translation of PTEN^19^. Taken together, our results indicate that the CPEB1–PTEN–AMPK–mTOR pathway mediates the beneficial effect of circRNA_002581 antagonization on autophagy restoration in NASH models.

There are several limitations of this study which should be acknowledged. First, it is better if we could confirm our findings in NASH human patients. Second, we found that a G-A point mutation at position 447 in the full-length sequence of circRNA_002581, which needs further study to investigate its role in NASH. Third, though our results supported autophagy as the key pathophysiological target of circRNA_002581 knockdown mediated NASH phenotype change, the detailed mechanism needs further study. For instance, previous study showed that mitochondrial-induced apoptosis could be inhibited by autophagy activation^38^, which is in accordance with our findings of reverse association between apoptosis and autophagy. Fourth, the CPEB1–PTEN–AMPK–mTOR pathway between circRNA_002581 knockdown mediated NASH phenotype change and autophagy seems too long and it would be more convincing if we could antagonize any step in this pathway and observe the change of both NASH phenotype and autophagy degree. In addition, previous study revealed the relationship between CPEB4 and ER stress, further studies should be carried out to explore the link between circRNA_002581 and ER stress in this model. Fifth, Though MCD diet is widely used in NASH study, it is better if we could further verify our findings in high fat-induced NASH model. Finally, since circRNAs are capable of regulating hundreds of miRNA–mRNA pairs, it is worth to investigate whether the observed changes of pro-inflammatory cytokine after circRNA_002581 knockdown is due to its unknown regulatory effect or just the accompanied phenomenon with NASH alleviation.

In summary, the present study provides new insights into the mechanism of NASH pathogenesis that circRNA_002581–miR-122–CPEB1 axis aggravates NASH partially through autophagy suppression, and also suggests that targeting circRNA_002581 may be a potential therapeutic strategy for NASH (Fig. 7c).

## Materials and methods

### Ethic statement

This study was carried out in accordance with the recommendation in the Guide for the Care and Use of Laboratory Animals of the National Institutes of Health. The protocol on animal was approved by the institutional review board of the First Affiliated Hospital of Zhejiang University. All mentioned cell lines in the followings were recently authenticated before purchasing and precluded with mycoplasma contamination before using.

### Establishing NASH model in in vitro and in vivo levels

A total of 44 male BALB/c mice aged 6 weeks were commercially purchased (Cavens Lab Animal, Suzhou, China) and randomly divided into four groups (each group *n* = 11): standard chow diet (SCD), MCD, MCD + control shRNA and MCD + circRNA_002581 shRNA. All mice received food and water ad libitum and were maintained on a 12/12 h light/dark cycle. SCD group was given a basic diet, while MCD group was given a MCD diet for consecutive 4 weeks as previously reported^39^. Meanwhile, the other two groups were, respectively, given tail vein injection of 5 × 10^8^ IU control (5′-TGGTCTAACCAGAGAGACCCAGTA-3′) and circRNA_002581 shRNA (5′-AACGGCCGTGTTTTGCTGTGC-3′) packaged Lentivirus 30 days (once per 5 days). Mice were sacrificed by neck dislocation at appointed time spot, where blood and liver tissue were collected for further analysis. NASH-like cell model was established by culturing AML-12 and NCTC-1469 cells (China Cell Culture Center, Shanghai, China) with HFFA, a mixture of oleate and palmitate at the final ratio of 2:1 and final concentration of 1 mM for 72 h.

### Transient transfection

HEK-293T cells (ATCC, USA) were transfected with circRNA_002581 overexpression plasmid or NC (empty vector), miR-122 mimics (sequence: AUCGAAUAGUCUGACUACAACUAAAAAA) or NC mimics (sequence: UCACAACCUCCUAGAAAGAGUAGA) using the Lipofectamine 2000 kit (Invitrogen, Shanghai, China) according to the manufacturer’s instructions for 48 h. NCTC-1469 cells were transfected with circRNA_002581 siRNA (sequence: AACGGCCGTGTTTTGCTGTGC) or corresponding scramble siRNA as NC (sequence: TGGTCTAACCAGAGAGACCCAGTA), miR-122 inhibitor (sequence: AUCGAAUACUCUGACUACAACU) or NC inhibitor (sequence: UCACAACCUCCUAGAAAGAGUAGA) using the Lipofectamine 2000 kit, and then treated with or without HFFA for 72 h.

### Metabolic measurements

After NASH mice and cell model establishment, serum and supernatant ALT and AST were tested with Hitachi 7600 clinical analyzer. Intracellular ROS level was assessed by flow cytometry using 2ʹ,7ʹ-dichlorodihydrofluorescein diacetate (DCFH-DA) (Sigma-Aldrich, USA). Briefly, after different treatment, each group of cells was incubated with DCFH-DA (10 µM) for 30 min in dark before termination of the experiment, then washed with serum-free medium and were determined using flow cytometry. H_2_O_2_ level was assessed using molybdenic acid-binding method (Nanjing Jiancheng Bioengineering Institue, Nanjing, China). ATP level was routinely detected as previously reported^40^.

### Full length amplification of circRNA_002581, gene sequencing verification and bioinformatics analysis

The genomic ID (mmu_circ_0001351) and NCBI sequence of circRNA_002581 were achieved from circbase (http://www.circbase.org). Initial PCR based on two backward-designed primers from the non-junction location of circRNA_002581 were carried out. The core circRNA_002581 sequence was achieved and compared with its official sequence in NCBI. Considering that such sequence may only represent the spliced instead of the full length sequence of circRNA_002581, additional five primers were designed, where their amplified sequences have partial overlaps. Such design could make sure the full length amplification of circRNA_002581 from the liver tissue of MCD-treated NASH mice. All primers are listed in Supplementary Table 2.

### Dual-Luciferase reporter gene analysis and cell transfection

The length of 3′UTR sequence of CPEB1 is 912 bp according to NCBI data. The pmirGLO vector (Promega, USA) was used to ligate the wild and mutant 3′UTR sequence of CPEB1 after double restriction enzyme cutting at the site of *Sac* I/*Sal* I (Supplementary Tables 3 and 4). The pmirGLO-CPEB1 wild and mutant 3′UTR were used to transfect competent cells with CaCl_2_ and cultured at 37 °C for 16 h, followed by vector extraction (TIANGEN, China) and verification by gene sequencing. The miR-122 mimics and NC mimics were synthesized and cotransfected HEK-293T cell (ATCC, USA) with pmirGLO-CPEB1 wild and mutant 3′UTR using lipofectamine 2000 (Invitrogen, USA). After culturing at 37 °C/5% CO_2_ for 24 h, those cotransfected cells were harvested and the luciferase activity was tested using a dual-luciferase reporter gene detection kit (Promega, USA). In this step, subjects were divided into five groups: blank cell control, pmirGLO-CPEB1 wild type + NC mimics, pmirGLO-CPEB1 wild type + miR-122 mimics, pmirGLO-CPEB1 mutant type + NC mimics, and pmirGLO-CPEB1 mutant type + miR-122 mimics.

### RNA-fluorescence in situ hybridization and RNA immunoprecipitation

RNA-FISH was routinely performed. Briefly, HEK-293T cell was separately transfected with plasmid over-expressing circRNA_002581 and circRNA_002581 + miR-122 mimics. These cells were cultured in coverslip and washed with CSK buffer (containing 0.5% TritonX-100 and 10 mM VRC), fixed with 4% paraformaldehyde at 4 °C for 10 min, sequentially dehydrated with 70%, 85% and 100% alcohol for each 5 min. Cells were attached on the glass slide and treated with the Cy5-coupled probe of circRNA_002581 (sequence: ACAACGGCCGTGTTTTGCTGTGCGTTCTCTGGG CTGGGTAGAGATG) and Cy3-coupled probe of miR-122 (sequence: TGGAGTGTG ACAATGGTGTT) at 37 °C overnight. They were then triply washed with 50% formamide at 42 °C for 5 min and re-dyed with DAPI and observed under fluorescence microscope.

RIP was performed with RIP-RNA-binding protein immunoprecipitation kit (merck millipore 17-700, USA)^41^. In detail, pre-equilibrated Protein-A Sepharose beads and 2 µg biotin antibody were mixed and incubate at 4 °C on horizontal shaker overnight. Second, RNA/binding protein complex was fixed from aspirated NIH3T3 cells from different groups after scraping, spinning, and washing with lysis buffer. Then the mixture of protein-A sepharose beads/antibody were added to the supernatant and incubated at 4 °C on horizontal shaker overnight. Finally, the mixture was spin down and sequentially re-suspended with protease K, TRIZOL, Chloroform, isopropanol, and alkaline phosphatase. The final mixture was dissolved in DEPC water, where the further extracted RNA was reverse transcribed and the expression of circRNA_002581 was detected. Sixteen pairs of PCR primers were designed to amplify circRNA_002581 (Supplementary Table 5).

### Hepatic and cellular TG measurement

Intrahepatic and intracellular TG contents were assayed using commercial kits (Applygen Technologies Inc., Beijing, China) according to the manufacturer’s advised protocol.

### Hematoxylin–eosin (H&E) and Oil red O staining

Mouse liver tissues were fixed in 10% neutral formalin, embedded in paraffin, sectioned, and stained with H&E for histological examination. NCTC-1469 cells in six-well plates were stained with Oil red O (Sigma-Aldrich, USA) to assess lipid accumulation in the cultured cells.

### Western blot, qRT-PCR, and cell apoptosis analysis

Routine western blot was used to detect the autophagy-related proteins (CPEB1, PTEN, AMPKα/p-AMPKα, mTOR/p-mTOR, LC3-I/II, p62) using an ECL chemiluminescence kit (Santa Cruz, USA) and GAPDH was used as internal control. Antibodies were supplied by Abcam (Cambridge, UK): GAPDH (ab8245); Cell Signaling Technology (Danvers, MA): CPEB1 (13583), PTEN (9559S), AMPK (5831S), p-AMPK (2535S), p-mTOR (2974S), mTOR (2983S); BD biosciences (New Jersey, USA): p62 (610497); MBL Beijing Biotech (Beijing, China): LC3 (PM036). For the mRNA analysis of miRNA and above-mentioned hepatic/cellular pro- inflammatory cytokines, 2 μg of retrieved total RNA was reversely transcribed using stem-loop antisense primer mix and AMV transcriptase (Takara, USA). U6 snRNA was amplified as a normalization control and the relative amount of each mRNA to U6 snRNA was calculated using the equation 2^−CT^, where CT = CTmiRNA-CTu6. PCR primers were designed (Supplementary Table 6). Cellular apoptosis was routinely analyzed using Annexin V-EGFP/PI double-staining method by flow cytometry according to the manufacturer’s instructions (Shanghai R&S Biotechnology Co., Ltd, China). Apoptosis in control and NASH animal models were carried out by routine TUNEL method (Roche, USA), where apoptosis index = (apoptotic cell/total cell)⁎100%.

### Autophagy evaluation by TEM, immunofluorescence, and autophagic flux test

Liver tissue was sequentially fixed in precooling 2.5% glutaral, cut into the size of 1 mm^3^ and thereafter routinely dehydrated, embedded, solidified, dyed, and observed under TEM. The number of hepatocellular autophagosome was calculated under every 10 TEM visual fields. The expression and localization of LC3-II in liver tissue and NCTC-1469 cell were routinely tested by laser confocal microscope. The autophagic flux test was carried out by adding 20 μM CQ as pretreatment to stop autophagy in cultured NCTC-1469 cells, followed by p62 level detection with western blot in different experimental sets.

### Statistics

Statistical analyses were performed using SPSS, version 16 (SPSS, Chicago, IL, USA). To ensure adequate animal model establishment, we initially (random number table method) allocated 11 mice into each group according to previous experience (considering the rate of mice death and the rate of successful model establishment). Since the data was not involving human beings, there was no blinding used in this experiment. The data are presented as means ± standard deviation (SD) when if were normally distributed or as medians if the distribution was skewed. Differences between groups were analyzed using Student’s *t*-test or the Mann–Whitney *U* test, where the equality of variance similarity was also compared. All experiments were performed in triplicate.

## Supplementary information

Supplementary Figure Legends

Supplementary Fig. 1

Supplementary Fig. 2

Supplementary Fig. 3

Supplementary Tables
