# Supplementary material for: Antagonizing circRNA_002581–miR-122–CPEB1 axis alleviates NASH through restoring PTEN–AMPK–mTOR pathway regulated autophagy
Source: Cell Death Dis. 2020 Feb 13;11(2):123. doi: 10.1038/s41419-020-2293-7 (PMC7018772; doi:10.1038/s41419-020-2293-7)
Supplement: Supplementary file 1 — Supplementary Figure Legends [file 41419_2020_2293_MOESM1_ESM.docx]

**Supplementary Figure Legends**

**Supplementary Fig. 1 Successful establishment of cells over-expressing circRNA_002581 and miR-122.** After transfecting circRNA_002581 and miR-122 in HEK-293T cells, their relative mRNA levels were both significantly increased. HEK-293T represents control cell; HEK-293T-NC represents HEK-293T cell transfected with vacant plasmid; HEK-293T-NC-circRNA_002581/miR-122 represents HEK-293T cell transfected with plasmid over-expressing circRNA_ 002581/miR-122 mimics. Error bars represent the SD. **p<0.01

**Supplementary Fig. 2 Selection of mice hepatocyte cell line for further study.** **a** After cultivation with HFFA, the TG levels were both significantly increased in AML-12 and NCTC-1469 cells. **b** The circRNA_002581 levels were both significantly increased in AML-12 and NCTC-1469 cells. **c** the miR-122 levels were both significantly decreased in AML-12 and NCTC-1469 cells. **d** The CPEB1 level was only significantly increased in NCTC-1469 cell. Error bars represent the SD. *p<0.05; **p<0.01.

**Supplementary Fig. 3 Antagonizing circRNA_002581-induced protective effect is mediated by miR-122 in vitro.** NCTC-1469 cells were transfected with circRNA_002581 siRNA or corresponding scramble siRNA as negative control, miR-122 inhibitor or negative control inhibitor, and then treated with HFFA for 72h. **a** Representative images of Oil Red O staining of NCTC-1469 cells (scale bar: 200 μm). **b** Relative mRNA levels of TNFα, IL-6, IL-1β **and** MCP-1 by quantitative real-time PCR. Error bars represent the SD. *p<0.05; **p<0.01.
