# Supplementary figures and images for: Antagonizing circRNA_002581–miR-122–CPEB1 axis alleviates NASH through restoring PTEN–AMPK–mTOR pathway regulated autophagy
Source: Cell Death Dis. 2020 Feb 13;11(2):123. doi: 10.1038/s41419-020-2293-7 (PMC7018772; doi:10.1038/s41419-020-2293-7)

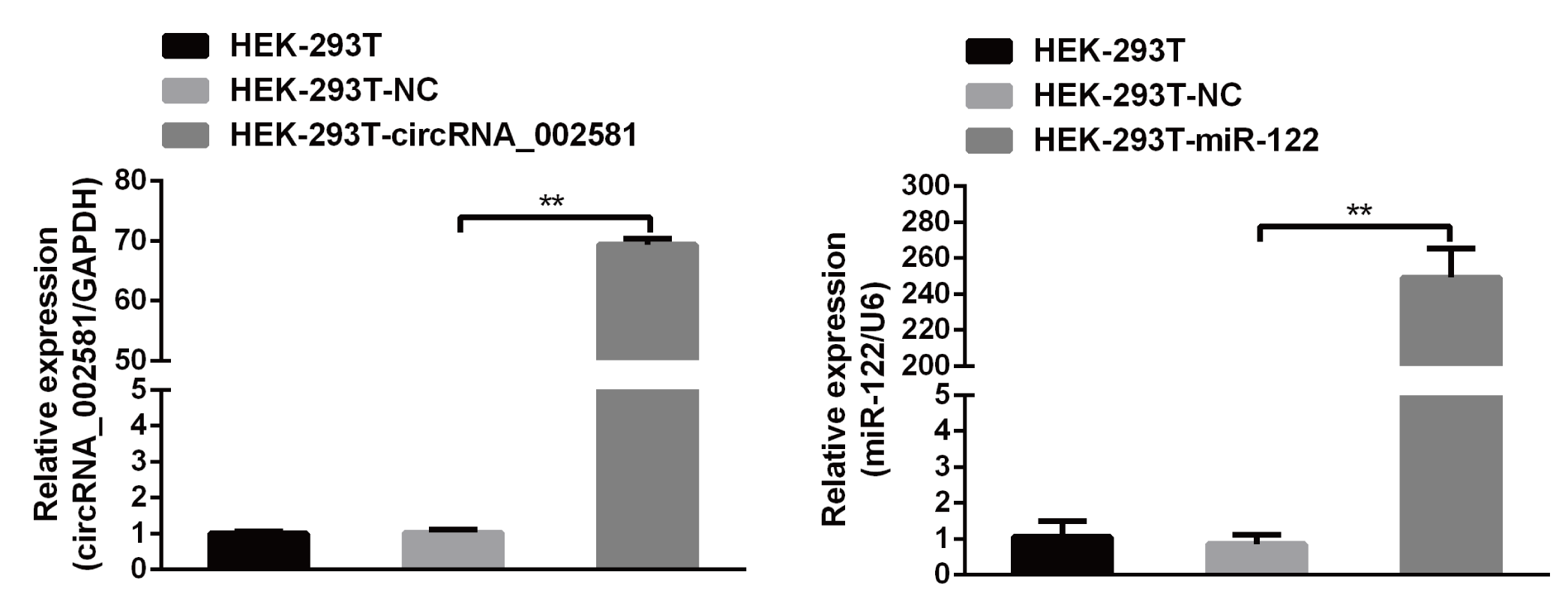

Supplement: Supplementary file 2 — Supplementary Fig. 1 [file 41419_2020_2293_MOESM2_ESM.tif]

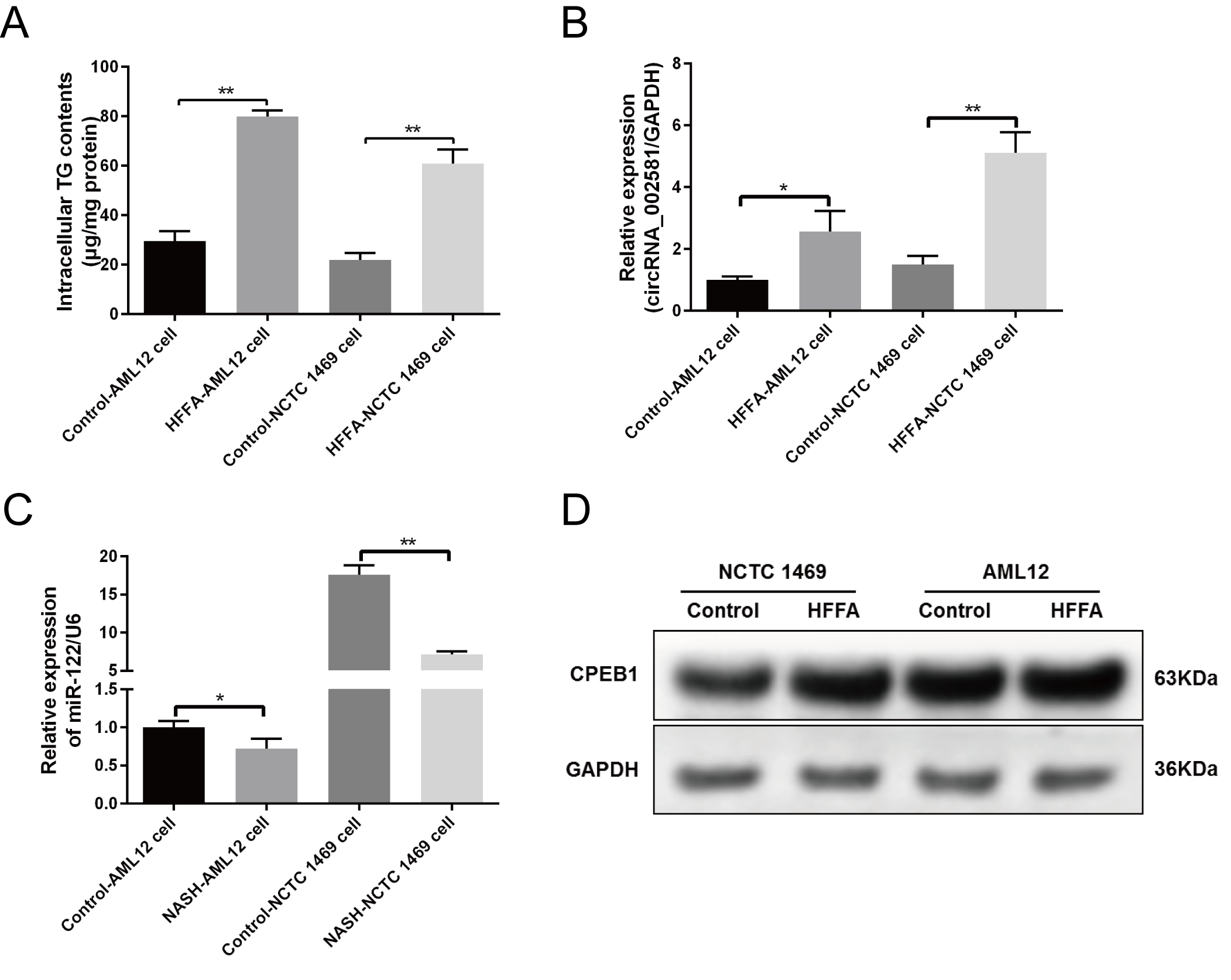

Supplement: Supplementary file 3 — Supplementary Fig. 2 [file 41419_2020_2293_MOESM3_ESM.tif]

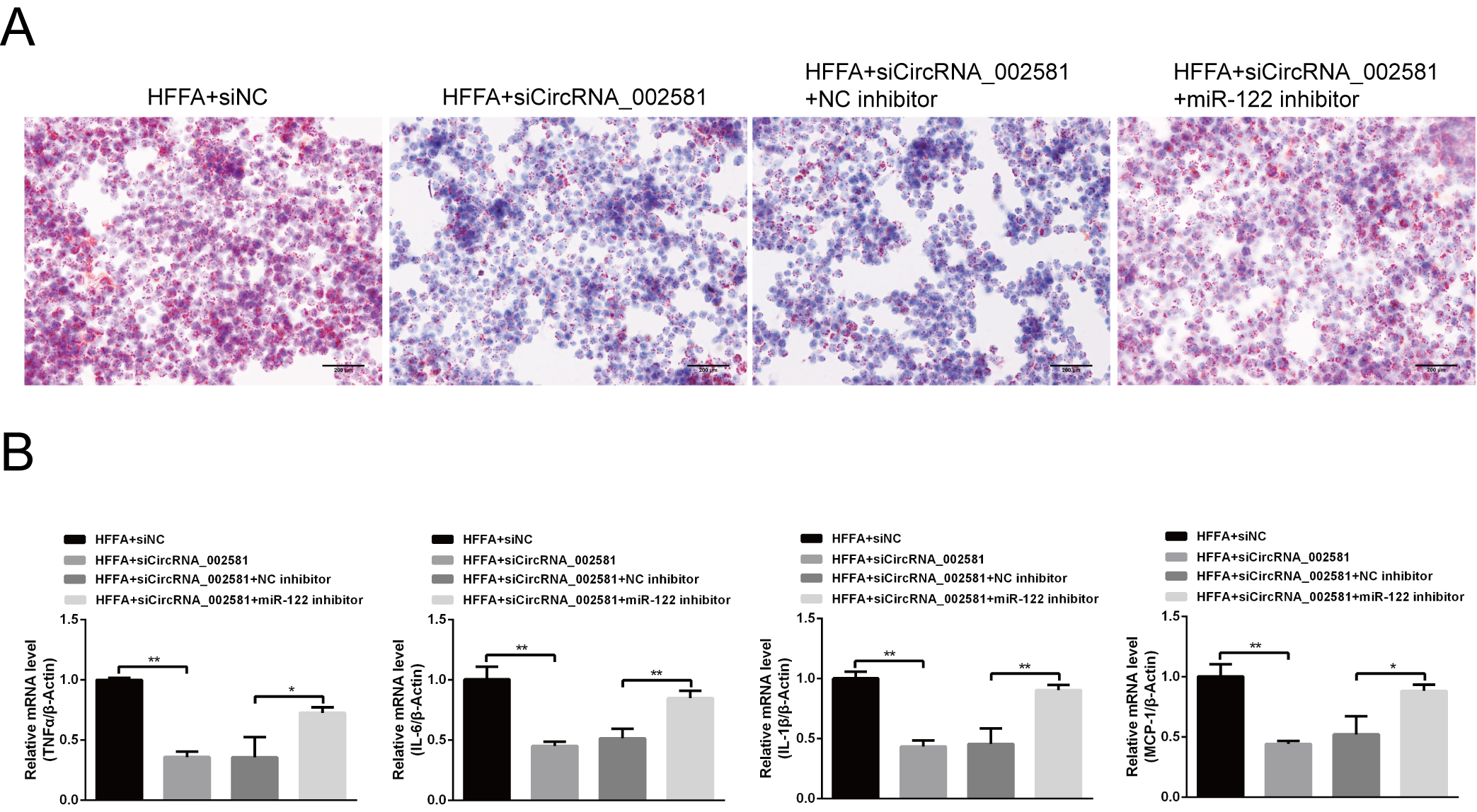

Supplement: Supplementary file 4 — Supplementary Fig. 3 [file 41419_2020_2293_MOESM4_ESM.tif]
