# Supplementary material for: Antagonizing circRNA_002581–miR-122–CPEB1 axis alleviates NASH through restoring PTEN–AMPK–mTOR pathway regulated autophagy
Source: Cell Death Dis. 2020 Feb 13;11(2):123. doi: 10.1038/s41419-020-2293-7 (PMC7018772; doi:10.1038/s41419-020-2293-7)
Supplement: Supplementary file 5 — Supplementary Tables [file 41419_2020_2293_MOESM5_ESM.docx]

**Supplementary Tables**

Supplementary Table 1, NAS score for different groups

| Group | NAS |
| --- | --- |
| SCD | 0.50±0.63 |
| MCD | 5.87±1.21** |
| MCD+ ctrl shRNA | 6.13±1.39** |
| MCD+ circRNA_002581 shRNA | 2.93±0.66^#^ |

**p<0.01, compared with SCD group; #p<0.05, compared with MCD group.

Supplementary Table 2, Primers for circRNA_002581 amplification and sequencing

Primers Sequence (5’-3’) Length (bp)

Initial

F GCTGTTTTGCTGTGCGTTCTCTGG 24

R CGGCCGTTGTCTCGGCCC 18

Additional

Cir-M-F TCATACCAAGAACCCACTCC 20

Cir-M-R CATCCGAATAGAAAGGAGTG 20

Cir-F GCAATGGAGAGACTTCACTCT 21

Cir-R GCAGACCCTGCAGCTGCCTT 20

Cir-M GAAGCATGGCAGAATACAGG 20

Supplementary Table 3, the whole sequence of pmirGLO-CPEB1 3’UTR wild type


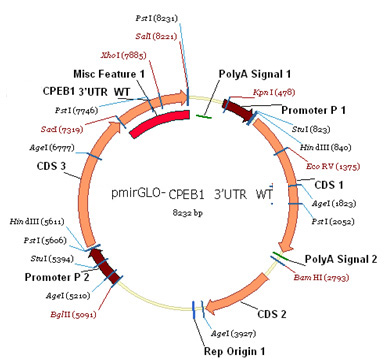


| 1  51  101  151  201  251  301  351  401  451  501  551  601  651  701  751  801  851  901  951  1001  1051  1101  1151  1201  1251  1301  1351  1401  1451  1501  1551  1601  1651  1701  1751  1801  1851  1901  1951  2001  2051  2101  2151  2201  2251  2301  2351  2401  2451  2501  2551  2601  2651  2701  2751  2801  2851  2901  2951  3001  3051  3101  3151  3201  3251  3301  3351  3401  3451  3501  3551  3601  3651  3701  3751  3801  3851  3901  3951  4001  4051  4101  4151  4201  4251  4301  4351  4401  4451  4501  4551  4601  4651  4701  4751  4801  4851  4901  4951  5001  5051  5101  5151  5201  5251  5301  5351  5401  5451  5501  5551  5601  5651  5701  5751  5801  5851  5901  5951  6001  6051  6101  6151  6201  6251  6301  6351  6401  6451  6501  6551  6601  6651  6701  6751  6801  6851  6901  6951  7001  7051  7101  7151  7201  7251  7301  7351  7401  7451  7501  7551  7601  7651  7701  7751  7801  7851  7901  7951  8001  8051  8101  8151 | CATGCAAGCT GATCCGGCTG CTAACAAAGC CCGAAAGGAA GCTGAGTTGG  CTGCTGCCAC CGCTGAGCAA TAACTAGCAT AACCCCTTGG GGCGGCCGCT  TCGAGCAGAC ATGATAAGAT ACATTGATGA GTTTGGACAA ACCACAACTA  GAATGCAGTG AAAAAAATGC TTTATTTGTG AAATTTGTGA TGCTATTGCT  TTATTTGTAA CCATTATAAG CTGCAATAAA CAAGTTAACA ACAACAATTG  CATTCATTTT ATGTTTCAGG TTCAGGGGGA GATGTGGGAG GTTTTTTTAA  GCAAGTAAAA CCTCTACAAA TGTGGTAAAA TCGAATTTTA ACAAAATATT  AACGCTTACA ATTTCCTGAT GCGGTATTTT CTCCTTACGC ATCTGTGCGG  TATTTCACAC CGCATACGCG GATCTGCGCA GCACCATGGC CTGAAATAAC  CTCTGAAAGA GGAACTTGGT TAGGTACCTT CTGAGGCGGA AAGAACCAGC  TGTGGAATGT GTGTCAGTTA GGGTGTGGAA AGTCCCCAGG CTCCCCAGCA  GGCAGAAGTA TGCAAAGCAT GCATCTCAAT TAGTCAGCAA CCAGGTGTGG  AAAGTCCCCA GGCTCCCCAG CAGGCAGAAG TATGCAAAGC ATGCATCTCA  ATTAGTCAGC AACCATAGTC CCGCCCCTAA CTCCGCCCAT CCCGCCCCTA  ACTCCGCCCA GTTCCGCCCA TTCTCCGCCC CATGGCTGAC TAATTTTTTT  TATTTATGCA GAGGCCGAGG CCGCCTCGGC CTCTGAGCTA TTCCAGAAGT  AGTGAGGAGG CTTTTTTGGA GGCCTAGGCT TTTGCAAAAA GCTTGATTCT  TCTGACACAA CAGTCTCGAA CCAAAGGCTG GAGCCACCAT GGCTTCCAAG  GTGTACGACC CCGAGCAACG CAAACGCATG ATCACTGGGC CTCAGTGGTG  GGCTCGCTGC AAGCAAATGA ACGTGCTGGA CTCCTTCATC AACTACTATG  ATTCCGAGAA GCACGCCGAG AACGCCGTGA TTTTTCTGCA TGGTAACGCT  GCCTCCAGCT ACCTGTGGAG GCACGTCGTG CCTCACATCG AGCCCGTGGC  TAGATGCATC ATCCCTGATC TGATCGGAAT GGGTAAGTCC GGCAAGAGCG  GGAATGGCTC ATATCGCCTC CTGGATCACT ACAAGTACCT CACCGCTTGG  TTCGAGCTGC TGAACCTTCC AAAGAAAATC ATCTTTGTGG GCCACGACTG  GGGGGCTTGT CTGGCCTTTC ACTACTCCTA CGAGCACCAA GACAAGATCA  AGGCCATCGT CCATGCTGAG AGTGTCGTGG ACGTGATCGA GTCCTGGGAC  GAGTGGCCTG ACATCGAGGA GGATATCGCC CTGATCAAGA GCGAAGAGGG  CGAGAAAATG GTGCTTGAGA ATAACTTCTT CGTCGAGACC ATGCTCCCAA  GCAAGATCAT GCGGAAACTG GAGCCTGAGG AGTTCGCTGC CTACCTGGAG  CCATTCAAGG AGAAGGGCGA GGTTAGACGG CCTACCCTCT CCTGGCCTCG  CGAGATCCCT CTCGTTAAGG GAGGCAAGCC CGACGTCGTC CAGATTGTCC  GCAACTACAA CGCCTACCTT CGGGCCAGCG ACGATCTGCC TAAGATGTTC  ATCGAGTCCG ACCCTGGGTT CTTTTCCAAC GCTATTGTCG AGGGAGCTAA  GAAGTTCCCT AACACCGAGT TCGTGAAGGT GAAGGGCCTC CACTTCAGCC  AGGAGGACGC TCCAGATGAA ATGGGTAAGT ACATCAAGAG CTTCGTGGAG  CGCGTGCTGA AGAACGAGCA GACCGGTGGT GGGAGCGGAG GTGGCGGATC  AGGTGGCGGA GGCTCCGGAG GGATTGAACA AGATGGATTG CACGCAGGTT  CTCCGGCCGC TTGGGTGGAG AGGCTATTCG GCTATGACTG GGCACAACAG  ACAATCGGCT GCTCTGATGC CGCCGTGTTC CGGCTGTCAG CGCAGGGGCG  CCCGGTTCTT TTTGTCAAGA CCGACCTGTC CGGTGCCCTG AATGAACTGC  AGGACGAGGC AGCGCGGCTA TCGTGGCTGG CCACGACGGG CGTTCCTTGC  GCAGCTGTGC TCGACGTTGT CACTGAAGCG GGAAGGGACT GGCTGCTATT  GGGCGAAGTG CCGGGGCAGG ATCTCCTGTC ATCTCACCTT GCTCCTGCCG  AGAAAGTATC CATCATGGCT GATGCAATGC GGCGGCTGCA TACGCTTGAT  CCGGCTACCT GCCCATTCGA CCACCAAGCG AAACATCGCA TCGAGCGAGC  ACGTACTCGG ATGGAAGCCG GTCTTGTCGA TCAGGATGAT CTGGACGAAG  AGCATCAGGG GCTCGCGCCA GCCGAACTGT TCGCCAGGCT CAAGGCGCGC  ATGCCCGACG GCGAGGATCT CGTCGTGACC CATGGCGATG CCTGCTTGCC  GAATATCATG GTGGAAAATG GCCGCTTTTC TGGATTCATC GACTGTGGCC  GGCTGGGTGT GGCGGACCGC TATCAGGACA TAGCGTTGGC TACCCGTGAT  ATTGCTGAAG AGCTTGGCGG CGAATGGGCT GACCGCTTCC TCGTGCTTTA  CGGTATCGCC GCTCCCGATT CGCAGCGCAT CGCCTTCTAT CGCCTTCTTG  ACGAGTTCTT CTGAGCGGGA CTCTGGGGTT CGAAATGACC GACCAAGCGA  CGCCCAACCT GCCATCACGA TGGCCGCAAT AAAATATCTT TATTTTCATT  ACATCTGTGT GTTGGTTTTT TGTGTGAATC GATAGCGATA AGGATCCTCT  TTGCGCTTGC GTTTTCCCTT GTCCAGATAG CCCAGTAGCT GACATTCATC  CGGGGTCAGC ACCGTTTCTG CGGACTGGCT TTCTACGTAA TGGTTTCTTA  GACGTCAGGT GGCACTTTTC GGGGAAATGT GCGCGGAACC CCTATTTGTT  TATTTTTCTA AATACATTCA AATATGTATC CGCTCATGAG ACAATAACCC  TGATAAATGC TTCAATAATA TTGAAAAAGG AAGAGTATGA GTATTCAACA  TTTCCGTGTC GCCCTTATTC CCTTTTTTGC GGCATTTTGC CTTCCTGTTT  TTGCTCACCC AGAAACGCTG GTGAAAGTAA AAGATGCTGA AGATCAGTTG  GGTGCACGAG TGGGTTACAT CGAACTGGAT CTCAACAGCG GTAAGATCCT  TGAGAGTTTT CGCCCCGAAG AACGTTTTCC AATGATGAGC ACTTTCAAAG  TTCTGCTATG TGGCGCGGTA TTATCCCGTA TTGACGCCGG GCAAGAGCAA  CTCGGTCGCC GCATACACTA TTCTCAGAAT GACTTGGTTG AGTACTCACC  AGTCACAGAA AAGCATCTTA CGGATGGCAT GACAGTAAGA GAATTATGCA  GTGCTGCCAT AACCATGAGT GATAACACTG CGGCCAACTT ACTTCTGACA  ACTATCGGAG GACCGAAGGA GCTAACCGCT TTTTTGCACA ACATGGGGGA  TCATGTAACT CGCCTTGATC GTTGGGAACC GGAGCTGAAT GAAGCCATAC  CAAACGACGA GCGTGACACC ACGATGCCTG TAGCAATGGC AACAACGTTG  CGCAAACTAT TAACTGGCGA ACTACTTACT CTAGCTTCCC GGCAACAATT  AATAGACTGG ATGGAGGCGG ATAAAGTTGC AGGACCACTT CTGCGCTCGG  CCCTTCCGGC TGGCTGGTTT ATTGCTGATA AATCTGGAGC CGGTGAGCGT  GGGTCTCGCG GTATCATTGC AGCACTGGGG CCAGATGGTA AGCCCTCCCG  TATCGTAGTT ATCTACACGA CGGGGAGTCA GGCAACTATG GATGAACGAA  ATAGACAGAT CGCTGAGATA GGTGCCTCAC TGATTAAGCA TTGGTAATTC  GAAATGACCG ACCAAGCGAC GCCCAACCGG TATCAGCTCA CTCAAAGGCG  GTAATACGGT TATCCACAGA ATCAGGGGAT AACGCAGGAA AGAACATGTG  AGCAAAAGGC CAGCAAAAGG CCAGGAACCG TAAAAAGGCC GCGTTGCTGG  CGTTTTTCCA TAGGCTCCGC CCCCCTGACG AGCATCACAA AAATCGACGC  TCAAGTCAGA GGTGGCGAAA CCCGACAGGA CTATAAAGAT ACCAGGCGTT  TCCCCCTGGA AGCTCCCTCG TGCGCTCTCC TGTTCCGACC CTGCCGCTTA  CCGGATACCT GTCCGCCTTT CTCCCTTCGG GAAGCGTGGC GCTTTCTCAT  AGCTCACGCT GTAGGTATCT CAGTTCGGTG TAGGTCGTTC GCTCCAAGCT  GGGCTGTGTG CACGAACCCC CCGTTCAGCC CGACCGCTGC GCCTTATCCG  GTAACTATCG TCTTGAGTCC AACCCGGTAA GACACGACTT ATCGCCACTG  GCAGCAGCCA CTGGTAACAG GATTAGCAGA GCGAGGTATG TAGGCGGTGC  TACAGAGTTC TTGAAGTGGT GGCCTAACTA CGGCTACACT AGAAGGACAG  TATTTGGTAT CTGCGCTCTG CTGAAGCCAG TTACCTTCGG AAAAAGAGTT  GGTAGCTCTT GATCCGGCAA ACAAACCACC GCTGGTAGCG GTGGTTTTTT  TGTTTGCAAG CAGCAGATTA CGCGCAGAAA AAAAGGATTT CAAGAAGATC  CTTTGATCTT TTCTACGGGG TCTGACGCTC AGTGGAACGA AAACTCACGT  TAAGGGATTT TGGTCATGAG ATTATCAAAA AGGATCTTCA CCTAGATCCT  TTTATAGTCC GGAAATACAG GAACGCACGC TGGATGGCCC TTCGCTGGGA  TGGTGAAACC ATGAAAAATG GCAGCTTCAG TGGATTAAGT GGGGGTAATG  TGGCCTGTAC CCTCTGGTTG CATAGGTATT CATACGGTTA AAATTTATCA  GGCGCGATTG CGGCAGTTTT TCGGGTGGTT TGTTGCCATT TTTACCTGTC  TGCTGCCGTG ATCGCGCTGA ACGCGTTTTA GCGGTGCGTA CAATTAAGGG  ATTATGGTAA ATCCACTTAC TGTCTGCCCT CGTAGCCATC GAGATAAACC  GCAGTACTCC GGCCACGATG CGTCCGGCGT AGAGGATCGA GATCTACCGG  GTAGGGGAGG CGCTTTTCCC AAGGCAGTCT GGAGCATGCG CTTTAGCAGC  CCCGCTGGGC ACTTGGCGCT ACACAAGTGG CCTCTGGCCT CGCACACATT  CCACATCCAC CGGTAGGCGC CAACCGGCTC CGTTCTTTGG TGGCCCCTTC  GCGCCACCTT CTACTCCTCC CCTAGTCAGG AAGTTCCCCC CCGCCCCGCA  GCTCGCGTCG TGCAGGACGT GACAAATGGA AGTAGCACGT CTCACTAGTC  TCGTGCAGAT GGACAGCACC GCTGAGCAAT GGAAGCGGGT AGGCCTTTGG  GGCAGCGGCC AATAGCAGCT TTGCTCCTTC GCTTTCTGGG CTCAGAGGCT  GGGAAGGGGT GGGTCCGGGG GCGGGCTCAG GGGCGGGCTC AGGGGCGGGG  CGGGCGCCCG AAGGTCCTCC GGAGGCCCGG CATTCTGCAC GCTTCAAAAG  CGCACGTCTG CCGCGCTGTT CTCCTCTTCC TCATCTCCGG GCCTTTCGAC  CTGCAGCCCA AGCTTGGCAA TCCGGTACTG TTGGTAAAGC CACCATGGAA  GATGCCAAAA ACATTAAGAA GGGCCCAGCG CCATTCTACC CACTCGAAGA  CGGGACCGCC GGCGAGCAGC TGCACAAAGC CATGAAGCGC TACGCCCTGG  TGCCCGGCAC CATCGCCTTT ACCGACGCAC ATATCGAGGT GGACATTACC  TACGCCGAGT ACTTCGAGAT GAGCGTTCGG CTGGCAGAAG CTATGAAGCG  CTATGGGCTG AATACAAACC ATCGGATCGT GGTGTGCAGC GAGAATAGCT  TGCAGTTCTT CATGCCCGTG TTGGGTGCCC TGTTCATCGG TGTGGCTGTG  GCCCCAGCTA ACGACATCTA CAACGAGCGC GAGCTGCTGA ACAGCATGGG  CATCAGCCAG CCCACCGTCG TATTCGTGAG CAAGAAAGGG CTGCAAAAGA  TCCTCAACGT GCAAAAGAAG CTACCGATCA TACAAAAGAT CATCATCATG  GATAGCAAGA CCGACTACCA GGGCTTCCAA AGCATGTACA CCTTCGTGAC  TTCCCATTTG CCACCCGGCT TCAACGAGTA CGACTTCGTG CCCGAGAGCT  TCGACCGGGA CAAAACCATC GCCCTGATCA TGAACAGTAG TGGCAGTACC  GGATTGCCCA AGGGCGTAGC CCTACCGCAC CGCACCGCTT GTGTCCGATT  CAGTCATGCC CGCGACCCCA TCTTCGGCAA CCAGATCATC CCCGACACCG  CTATCCTCAG CGTGGTGCCA TTTCACCACG GCTTCGGCAT GTTCACCACG  CTGGGCTACT TGATCTGCGG CTTTCGGGTC GTGCTCATGT ACCGCTTCGA  GGAGGAGCTA TTCTTGCGCA GCTTGCAAGA CTATAAGATT CAATCTGCCC  TGCTGGTGCC CACACTATTT AGCTTCTTCG CTAAGAGCAC TCTCATCGAC  AAGTACGACC TAAGCAACTT GCACGAGATC GCCAGCGGCG GGGCGCCGCT  CAGCAAGGAG GTAGGTGAGG CCGTGGCCAA ACGCTTCCAC CTACCAGGCA  TCCGCCAGGG CTACGGCCTG ACAGAAACAA CCAGCGCCAT TCTGATCACC  CCCGAAGGGG ACGACAAGCC TGGCGCAGTA GGCAAGGTGG TGCCCTTCTT  CGAGGCTAAG GTGGTGGACT TGGACACCGG TAAGACACTG GGTGTGAACC  AGCGCGGCGA GCTGTGCGTC CGTGGCCCCA TGATCATGAG CGGCTACGTT  AACAACCCCG AGGCTACAAA CGCTCTCATC GACAAGGACG GCTGGCTGCA  CAGCGGCGAC ATCGCCTACT GGGACGAGGA CGAGCACTTC TTCATCGTGG  ACCGGCTGAA GAGCCTGATC AAATACAAGG GCTACCAGGT AGCCCCAGCC  GAACTGGAGA GCATCCTGCT GCAACACCCC AACATCTTCG ACGCCGGGGT  CGCCGGCCTG CCCGACGACG ATGCCGGCGA GCTGCCCGCC GCAGTCGTCG  TGCTGGAACA CGGTAAAACC ATGACCGAGA AGGAGATCGT GGACTATGTG  GCCAGCCAGG TTACAACCGC CAAGAAGCTG CGCGGTGGTG TTGTGTTCGT  GGACGAGGTG CCTAAAGGAC TGACCGGCAA GTTGGACGCC CGCAAGATCC  GCGAGATTCT CATTAAGGCC AAGAAGGGCG GCAAGATCGC CGTGTAATTC  TAGTTGTTTA AACGAGCTCG ATTCCAGCTA GAGGAGCTGG CCTTGCCCAG  TGTCCTGTGG TGCCAAAGGC TGTCAAGTCA GGCAGCAACT CGCTGCACAG  GCGCCATGGG AGCTAGCTTT GCGCAGACAA GGGAGAATCG TAGTCACCTT  TGTACTTGCT AACTCTGTCT TTGTTTCTGC ACTAATTAAT GCACAATGAG  TTTTGTCAGG TCTTGTTTTC AGTGGGGTGT GCCAGAGCAA GGACCCTCGG  CTCACCCTCA AGCAATTGTA GTTTTCCCAG ATTCTAGTTC CTCATTTTGC  AAATGAAAAT AACAACAACA ACCACTGTGT CCAGAAGGTC TGACACAGAT  GCCTACACTT GGGTTTATTT ATTAAAGCGC TTTTTACATT CCTTGCAATA  CTGATGGTGG TGATGCGCAG GTCTCATTGG TTCGTTCATT CTGCAGTTGC  CATACAGTGC CTTTCCATTG ATTTAACCCC CACCTGAACG GCATCAATTG  AGTGTTCAGC TGGTGTTTTT TACTGTAACA AACAAAGGAG ACTTTGCTCT  TCATTTAAAC CAAATCATAT TTCATAGTTT ACGCTCGAGG GTTTTTACTG  GTTCCTTTTT ACACTCCTTA AAACAGTTTT TAAGTCGTTT GGAACAATAT  ATTTTTTTTT CTTTCTTGGC AGCTTTTAAC ATTATAGCAA ATTTGTGTCT  GGGGGACTGC TGGTCACAGT TGCAAATCAA AGCATTTGTA ACCAAGAGAA  AAATTATTTT ATTTAAAACT GGACCGGAGG AAAAGTCTGA GCAGCTGCTG  TATATAGTTT TAAATGGTTT GTGGCACCTT ATGTTGCACT TATGTTGGGG  GAGGGTTGAT AGAAGTTTTT AATCACAGTC ACAGGACTTT TTCTTTTGTA  ACTGAGCTTA AAAATTAAAG TCGACCTGCA GG |
| --- | --- |

The sequence from yellow color covered area is the inserted 3’-UTR of CPEB1, where eight red letters represent the sequence for mutation.

Supplementary Table 4, the sequence of CPEB1 3’UTR mutant type

TAGTTGTTTA AACGAGCTCG ATTCCAGCTA GAGGAGCTGG CCTTGCCCAG

TGTCCTGTGG TGCCAAAGGC TGTCAAGTCA GGCAGCAACT CGCTGCACAG

GCGCCATGGG AGCTAGCTTT GCGCAGACAA GGGAGAATCG TAGTCACCTT

TGTACTTGCT AACTCTGTCT TTGTTTCTGC ACTAATTAAT GCACAATGAG

TTTTGTCAGG TCTTGTTTTC AGTGGGGTGT GCCAGAGCAA GGACCCTCGG

CTCACCCTCA AGCAATTGTA GTTTTCCCAG ATTCTAGTTC CTCATTTTGC

AAATGAAAAT AACAACAACA ACCACTGTGT CCAGAAGGTC TGACACAGAT

GCCTACACTT GGGTTTATTT ATTAAAGCGC TTTTTACATT CCTTGCAATA

CTGATGGTGG TGATGCGCAG GTCTCATTGG TTCGTTCATT CTGCAGTTGC

CATACAGTGC CTTTCCATTG ATTTAACCCC CACCTGAACG GCATCAATTG

AGTGTTCAGC TGGTGTTTTT TACTGTAACA AACAAAGGAG ACTTTGCTCT

TCATTTAAAC CAAATCATAT TTCATAGTTT ACGCTCGAGG GTTTTTACTG

GTTCCTTTTT CTCTGTTGTA AAACAGTTTT TAAGTCGTTT GGAACAATAT

ATTTTTTTTT CTTTCTTGGC AGCTTTTAAC ATTATAGCAA ATTTGTGTCT

GGGGGACTGC TGGTCACAGT TGCAAATCAA AGCATTTGTA ACCAAGAGAA

AAATTATTTT ATTTAAAACT GGACCGGAGG AAAAGTCTGA GCAGCTGCTG

TATATAGTTT TAAATGGTTT GTGGCACCTT ATGTTGCACT TATGTTGGGG

GAGGGTTGAT AGAAGTTTTT AATCACAGTC ACAGGACTTT TTCTTTTGTA

ACTGAGCTTA AAAATTAAAG TCGACCTGCA GG

The mutant 3’UTR of CPEB1 is shown in yellow color covered area. The insertion area of pmirGLO is identical with the wild type. Eight red letters represent the mutated sequence comparing with wild type.

Supplementary Table 5, PCR primer sequence for circRNA-002581

| Primers | Sequence (5’-3’) | Length (bp) |
| --- | --- | --- |
| 1-F | GGCAAAGACATGTACCTGAGCC | 22 |
| 1-R | CCAGAGAACGCACAGCAA | 18 |
| 2-F | GCTGGGTAGAGATGGCCGAG | 20 |
| 2-R | TGCTGCCCTTCCACGGGT | 18 |
| 3-F | GCTACTTTCCTGTATTCTGCCATGC | 25 |
| 3-R | GGGATGTGGTGGGGTTAG | 18 |
| 4-F | ACTCAGAAGCACGCCCCTAAG | 21 |
| 4-R | ACTGTTAATAGGTGGACA | 18 |
| 5-F | TTGCGGGGAAACAAACTATGC | 21 |
| 5-R | TAAAAGTGAGACAACACA | 18 |
| 6-F | ATAAAGTGAGTCTACTTT | 18 |
| 6-R | CCCTGCCTGATGTATTCT | 18 |
| 7-F | AAAGCTGTATTCAAGCTCTGTGGC | 24 |
| 7-R | TCATGATGTACGGCTGGG | 18 |
| 8-F | TGAAAAGCCAGCTCTCAG | 18 |
| 8-R | GTCTCAACAAGGGTTTTC | 18 |
| 9-F | TTCTCACTTCCTCCAGGGGGAGG | 23 |
| 9-R  10-F  10-R  11-F | CCCCCGCTGAGGAGGGGA  TTTACACTAGTATGATTG  CTGAGAGCCTCCCCTCCA  CCATCCTGAGTCCCAGCTGTAG | 18  18  18  22 |
| 11-R | GGACAGATCAGTTGCTCT | 18 |
| 12-F | CTTTGGGATGGGAGCCTCAC | 20 |
| 12-R | TCAAACACATTTTTAAGG | 18 |
| 13-F | GGACTGTACGGGGGAAGTTACATA | 24 |
| 13-R | TGATCTGAGGCATGTAAT | 18 |
| 14-F | TGGGCAGACACTTAGCCC | 18 |
| 14-R | AGCTCACTTCCCTGTGCT | 18 |
| 15-F | GCCTGCCGTCTGTACAGA | 18 |
| 15-R | GGTCCCCCTCACTATTCT | 18 |
| 16-F | TGAGGGGTGGTAGCTCAT | 18 |
| 16-R | CTGGGAGCCAAGGAGAAA | 18 |
| GAPDH-F | GTTACCAGGGCTGCCTTCTC | 20 |
| GAPDH-R | GGGTTTCCCGTTGATGACC | 19 |

Supplementary Table 6, PCR primer sequence for pro-inflammatory cytokines

| Primers | Sequence (5’-3’) | Length (bp) |
| --- | --- | --- |
| TNFα-F | CCCTCACACTCAGATCATCTTCT | 23 |
| TNFα-R | GCTACGACGTGGGCTACAG | 19 |
| IL-6-F | TAGTCCTTCCTACCCCAATTTCC | 23 |
| IL-6-R | TTGGTCCTTAGCCACTCCTTC | 21 |
| IL-1β-F | GCAACTGTTCCTGAACTCAACT | 22 |
| IL-1β-R | CCCTCACACTCAGATCATCTTCT | 23 |
| MCP-1-F | GCAGGTCCCTGTCATGCTTC | 20 |
| MCP-1-R | GAGTGGGGCGTTAACTGCAT | 20 |
| β-Actin | CACGATGGAGGGGCCGGACTCATC | 24 |
| β-Actin | TAAAGACCTCTATGCCAACACAGT | 24 |
